# Cascade Monitoring in Multidisciplinary Adolescent HIV Care Settings: Protocol for Utilizing Electronic Health Records

**DOI:** 10.2196/11185

**Published:** 2019-05-30

**Authors:** Amy L Pennar, Tyra Dark, Kit N Simpson, Sitaji Gurung, Demetria Cain, Carolyn Fan, Jeffrey T Parsons, Sylvie Naar

**Affiliations:** 1 College of Medicine Florida State University Tallahassee, FL United States; 2 Department of Healthcare Leadership and Management Medical University of South Carolina Charleston, SC United States; 3 Center for HIV Educational Studies and Training Hunter College City University of New York New York, NY United States; 4 Hunter College Department of Psychology Hunter College City University of New York New York, NY United States; 5 Health Psychology and Clinical Science Doctoral Program Graduate Center City University of New York New York, NY United States

**Keywords:** HIV treatment cascade, electronic health records, youth living with HIV, HIV, treatment or care adherence, youth

## Abstract

**Background:**

Past research shows that youth living with HIV (YLH) are not as engaged in the HIV treatment cascade as other HIV-positive populations. To achieve the health benefits of rapid and widespread testing and advanced pharmacologic treatment, YLH must be fully engaged in every stage of the treatment cascade. Cascade monitoring provides an opportunity to assess the youth care cascade, including engagement in care and when youth commonly drop out of care, across 10 clinical sites in the United States. Collecting electronic health record (EHR) data for prevention and care across participant recruitment venues within the Adolescent Medicine Trials Network (ATN) allows for monitoring of the prevention and care cascades within the ATN, for comparing the ATN population to large-scale surveillance, for future integration of technology-based interventions into EHRs, and for informing ATN strategic planning.

**Objective:**

The aim of this protocol study is to examine the trends in treatment cascade, including whether patients are receiving antiretroviral therapy, adhering to regimens, attending care appointments, and maintaining suppressed viral loads, to guide new protocol development and to facilitate community engagement. This protocol is part of the ATN Scale It Up (SIU) program described in this issue.

**Methods:**

Deidentified EHR data of YLH, aged 15 to 24 years, will be collected annually (2017 to 2022) from 10 ATN clinical sites, resulting in patient data from 2016 to 2021. These data will be transferred and stored using Dropbox Business, a Health Insurance Portability and Accountability Act–compliant site and then analyzed by the SIU analytic core.

**Results:**

This study was launched in December 2017 in 10 clinical sites, with 2016’s EHR data due on January 31, 2017. All 10 sites electronically uploaded their EHR data. The mandatory variables requested to monitor cascade of care include date of visit, age, gender, height, weight, race, ethnicity, viral load, and International Classification of Diseases codes for other diagnosis. In total, 70% of the sites provided data for all mandatory variables. The remaining mandatory variables were manually extracted.

**Conclusions:**

This study will provide a platform to determine how YLH across the nation progress through or drop out of the HIV treatment cascade. It will also provide a foundation for assessing impact of SIU projects on treatment cascade outcomes.

**International Registered Report Identifier (IRRID):**

DERR1-10.2196/11185

## Introduction

### Background

In the United States, youth living with HIV (YLH)—especially ethnic and racial minority youth and men who have sex with men (MSM)—are experiencing disproportionately high rates of morbidity [1], which places them at risk for early mortality. Despite the fact that dramatic decreases in HIV transmission are achievable with currently available treatments and interventions (such as antiretroviral treatment [ART], pre-exposure prophylaxis [PrEP], and rapid and widespread testing), such decreases have not been realized among youth. Only 42% of YLH aged 13 to 24 years are aware of their sero-status, compared with 83.5% of all HIV-positive Americans [2].

Therefore, it is clear that the HIV health care systems are failing to engage youth at risk for HIV and YLH at one or more steps of the cascade. The HIV prevention cascade includes routine HIV and sexually transmitted infection (STI) testing, as well as PrEP knowledge, access, uptake, and adherence when warranted [3-5]. The HIV treatment cascade describes care for those living with HIV, and includes diagnosis, linkage to care, timely initiation of care, persistence and adherence to ART, and sustained viral suppression.

Even when youth enter the treatment cascade through diagnosis, knowledge of HIV status does not necessarily result in linkage to care. Compared with 75% of adults, less than two-thirds of YLH are linked to care within 6 to 12 months after diagnosis [6]. Importantly, even linkage to care does not guarantee quality or effective care. The physician must initiate ART early, even if there is erratic behavior on the part of the youth, and YLH must recognize that ART is a lifelong commitment and requires a high degree of adherence. Even with appropriate ART initiation and adherence, drug resistance is present (even at baseline) in approximately 10% of youth [7-9]. Although effectiveness trials report viral suppression rates of 80% or more among YLH receiving ART, observational studies (ie, real-world occurrence) find much lower rates, closer to 50% [9]. Zanoni and Mayer presented the HIV care cascade of care for adolescent and young adults in the United States with declining numbers for those diagnosed, engaged in medical care, initiated antiretroviral therapy, and achieved viral suppression [9].

Ultimately, the goal of those involved in HIV prevention and care is to achieve maximum commitment to the HIV prevention and treatment cascades. Mathematical modeling indicates that even with 90% detection of HIV infections, followed by 90% engagement in care of YLH, 90% appropriate treatment of those in care, and 90% viral suppression of treated individuals, approximately 34% of YLH will remain viremic (ie, presence of a virus in the blood) [3]. In summary, implementation of sustainable service delivery interventions along multiple points in the cascade is necessary to achieve maximal benefits of increased access to ART and increase viral suppression [10].

To achieve sustained viral suppression, a youth must determine their HIV status and effectively engage in points along the HIV treatment cascade. Engagement should begin at the prevention cascade level, in that youth should have routine HIV testing and consider postexposure prophylaxis or PrEP if warranted. If HIV positive, the youth must immediately engage with the care system, initiate ART and comprehend and embrace the necessity of proper adherence, be retained in care, and maintain viral suppression. Collectively, these steps comprise proper HIV and sexual health self-management, defined as “strategies to help individuals...and their caregivers better understand and manage their illness and [/or] improve their health behaviors” [11]. Every step of the HIV treatment cascade requires at-risk youth or YLH to make decisions to engage with the system or to modify their behaviors, that is, every step requires active *self-management*. In fact, large numbers of at-risk youth are not seeking HIV/STI testing or other prevention services; if positive, they are not engaging in care. Even when they are in care, many youths are not sustaining adequate HIV care and treatment. They may be engaging in other risky behaviors, such as substance use, that interfere across all points in the cascade.

The Scale It Up (SIU) Research Projects [12] were funded as part of the Adolescent Medicine Trials Network (ATN) and designed to advance the field of implementation science by employing 3 types of effectiveness implementation hybrid designs, all addressing self-management and inner and outer context variables including sociopolitical culture, organizational characteristics, culture and climate, leadership, dynamics of the multidisciplinary team, facilitator characteristics and attitudes, training fidelity monitoring and support, efforts, intervention fit, and fiscal viability that are involved in successful implementation. SIU utilizes motivational interviewing (MI) to provide a clear framework for improving patient-provider communication and promoting behavior change (ie, improved self-management) using client-centered methods for enhancing motivation and self-efficacy [13]. These are provider-driven strategies to meet patients where they are currently at regarding behaviors and build motivation for increased change. High-quality patient-provider relationships are associated with a greater likelihood of patients’ receiving ART, ART adherence, attending appointments, and having lower viral load [14-18]. Through enhancing implementation of treatment protocols and patient-provider relationships, SIU focuses on improving youths’ outcomes along the prevention and care cascades. Examining trends in the care cascade is therefore critical for providing a foundation for outcomes assessment across SIU projects.

Cascade monitoring (CM) ATN 154 is a center-wide protocol in SIU focusing on the HIV care cascade as well as health factors (eg, cardiovascular functioning) known to be consequential for the long-term health outcomes of individuals living with HIV. Information collected in CM will monitor impact from other SIU protocols on potential gains in the treatment cascade. CM specifically collects only data that can be extracted from electronic health records (EHRs); therefore, CM does not include HIV prevention data, as the participating clinical sites generally did not have prevention data available for extraction. Documenting trends in the care cascade will facilitate a deeper comprehension of when in the care cascade patients most commonly drop out, and what populations commonly disengage from the cascade, thereby providing researchers, clinicians, and community stakeholders a pulse on the adolescent HIV epidemic. Furthermore, the use of electronically extracted variables that measure general patient conditions by International Statistical Classification of Diseases and Related Health Problems-10th revision (ICD-10) codes, as well as cascade-specific variables, enables us to identify clusters of data points that are meaningful for monitoring differences in treatment cascade retention. These clusters of EHR variables, called informatics *EHR phenotypes*, can then be used to identify similar patients in other electronic data systems. The EHR phenotypes identified for YLH through CM will be used in the cost-effectiveness analysis for the SIU studies and used with large archival databases to estimate the prevalence of cascade failures to YLH in other community settings not included in the CM study.

### Aims

CM provides an opportunity to assess trends to guide new protocol development and to facilitate community engagement. These data will support the Adolescent Medicine Trials Network (ATN) to have a finger on the pulse of the epidemic and will provide feedback to sites and community partners. Using the EHR phenotypes from CM, the patterns observed in the 10 SIU clinical sites can be extended to identify YLH in other settings, which can help policymakers and service providers improve systems and services and address barriers to care to better support individuals as they move from one step in the HIV care continuum to the next.

- Aim 1: Develop a common data model of variables of relevance for measuring cascade outcomes across 10 multidisciplinary adolescent HIV clinics and implement EHR data extraction (downloading) annually for 2016 to 2021 calendar years.
- Aim 2: Utilize data submission reports to identify a systematic EHR data extraction process to be utilized by clinic sites.
- Aim 3: Using extracted EHR data, determine how many of those retained in care and receiving ART keep clinic appointments and achieve viral suppression.
- Aim 4A: Determine when patients most commonly drop out of the HIV care continuum, and what populations commonly do so, in the SIU clinical sites.
- Aim 4B: Develop a phenotype for HIV-infected adolescents from the EHR CM data. Use the phenotypes applied to large national datasets to estimate variations in rates of cascade failure for YLH in the United States to help policymakers and service providers improve systems and services to better support individuals as they move from one step in the HIV care continuum to the next.
- Aim 5: Measure the effect of clinic-based interventions in SIU on longitudinal HIV care cascade outcomes across the 10 multidisciplinary adolescent HIV care clinics (approximately 1200 patients). This center-wide protocol will support the aims of the separate SIU projects.
- Aim 6: Apply the phenotype for HIV-infected adolescents from the EHR CM data to national databases to estimate costs weights for use in the assessment of cost-effectiveness of the SIU interventions.

## Methods

### Overview

This study (CM, ATN 154) is part of the SIU program as described in the overview paper in this issue [12]. Deidentified EHR data will be collected retrospectively from 10 clinical sites, also known as Subject Recruitment Venues (SRVs), participating in the SIU. The sites are as follows:

1. Site 1: Baltimore—Johns Hopkins University, Maryland.
2. Site 2: Birmingham—University of Alabama at Birmingham, Alabama.
3. Site 3: Brooklyn—SUNY Downstate Medical Center, New York.
4. Site 6: Los Angeles—Children’s Hospital Los Angeles, California.
5. Site 7: Memphis—St. Jude Children’s Research Hospital, Tennessee.
6. Site 8: Miami—University of Miami, Florida.
7. Site 10: Philadelphia—Children’s Hospital of Philadelphia, Pennsylvania.
8. Site 11: San Diego—University of California San Diego, California.
9. Site 12: Tampa—University of South Florida, Florida.
10. Site 13: Washington, DC—Children’s National Health System, District of Columbia.

**Figure 1:**
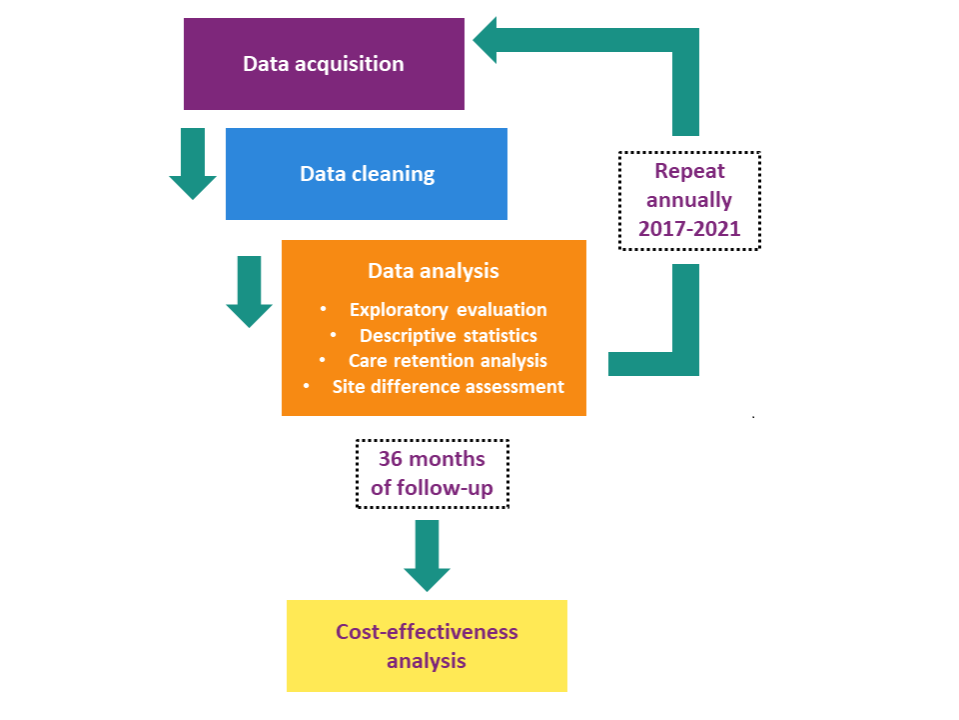
Cascade monitoring study design.

The first extracts will contain standard of care and treatment visits in the full year of 2016 and associated data (see Table 1) for all YLH, aged 15 to 24 years, treated at sites. Subsequently, 1-year data extracts will be requested from sites annually, with the final year of data uploaded in 2022 for the full year of 2021 (see Figure 1).

### Data Acquisition

Data collection will occur at all SIU clinical sites providing care to YLH by site personnel. The scientific and leadership teams have determined that there will not be resources allotted to do chart review for the CM measures; therefore, the CM protocol team will receive only measures available for extraction from the clinics’ electronic data systems. The provision of electronically downloaded data, relative to hand extracted, also reduces the risk of data entry error, particularly for complex variables (eg, ICD-10 codes). However, given that longitudinal surveillance of viral load is critical to the success of the protocol, hand-extracted viral load data are the only exception when it cannot be electronically downloaded. All data transferred by the sites to the CM protocol team will be deidentified at the sites before data transfer. Having the sites create and update a master list of patients and unique identifiers is crucial for conducting longitudinal data analysis and following patient attributes and outcomes over time. Sites will be provided with the instructions on the unique identifiers that should be used to create participant identification numbers.

**Table 1:** Measures.

Type and variable		Metrics and notes	
**Time varying**			
	Patient ID		Unique to study, should not match patient’s medical record file (ie, deidentified); see table below
	Visit date		MM/DD/YYYY
	Antiretroviral Medications—Name		Name of medication; include all prescribed
	Viral load results		Quantitative value (parts per cubic ml)
	CD4 cell count result		Absolute count
	Height		Meters
	Weight		Kg
	Blood pressure—systolic		mmHg
	Blood pressure—diastolic		mmHg
	Cholesterol panel—total		mg/dL
	Cholesterol panel—low density lipoprotein		mg/dL
	Cholesterol panel—high density lipoprotein		mg/dL
	Cholesterol panel—triglycerides		mg/dL
	Blood glucose—hemoglobin A_1c_		mg/dL
	Blood glucose—fasting		mg/dL
	Tobacco use—current status		current/past/never
	Tobacco use—how much		Number of packs per day
	Tobacco use—how long		Number of years
	Chlamydia test result		Positive/negative OR reactive/nonreactive; if titer please enter positive or reactive
	Gonorrhea test result		Positive/negative OR reactive/nonreactive; if titer please enter positive or reactive
	Herpes test result		Positive/negative OR reactive/nonreactive; if titer please enter positive or reactive
	Syphilis test result		Positive/negative OR reactive/nonreactive; if titer please enter positive or reactive
	Human papilloma virus test result		Positive/negative OR reactive/nonreactive; if titer please enter positive or reactive
	Other non-HIV medication—Name		Name of medication; include all prescribed
	Patient zip code		Last known/documented
	International Statistical Classification of Diseases and Related Health Problems-10th revision Diagnoses Codes		Include all from visit; extracted as left-hand justified character variables
	CPT^a^-4 Procedure Codes		Include all from visit; extracted as 5-digit numerical variables
	Health care common procedure coding system Procedure Codes		Include all from visit; if used instead of CPT codes; extracted as 5-digit character variable
**Time invariant**			
	Patient ID		Unique to study, should not match patient’s medical record file (ie, deidentified)
	Current age		Age as of January 1 of the year of data download (eg, 01/01/2016 for 2016 data download)
	Age at HIV diagnosis		Years
	Date of HIV diagnosis		MM/DD/YYYY
	Gender		Self-report; for example, male, female, transgender male-to-female, transgender female-to-male, other
	Race		Self-report
	Ethnicity		Hispanic/non-Hispanic/other as available /self-report
	Sexual-orientation		Self-report
	Mode of transmission		For example, men who have sex with men, intravenous drug use, high-risk heterosexual
	Date of entry into care		MM/DD/YYYY
	Date of death (if applicable)		MM/DD/YYYY

^a^CPT: Current Procedural Terminology.

The SIU Recruitment and Enrollment Center (REC) is a subsidiary of the management core and works with the CM protocol team to communicate and work with SRVs to obtain EHR data and troubleshoot challenges. The REC issues launch materials including example templates to sites before download, specifying data elements, formats, and ordering. Data structure will likely vary by site and depend on the resources and capabilities of each site. The REC is equipped to process various data structures and formats, in this case.

The CM protocol requires data to be electronically downloaded, not hand-extracted. If a site cannot, at a minimum, download viral load, date of appointment, age, height, weight and ICD-10 diagnoses codes, then the CM protocol team will consider dropping the site. The site needs to discuss with their information technology team/department to determine if downloading is possible, and any associated cost. The CM protocol team will then determine whether it is feasible for the site to remain as part of CM and, if applicable, an acceptable date of data transfer.

EHR data on cost-effectiveness variables include ICD-10 diagnoses codes (extracted as left-hand justified character variables), current procedural terminology (version 4) procedure codes (extracted as 5-digit numerical variables), and health care common procedure coding system procedure codes (if used instead of CPT codes; extracted as 5-digit character variables). A data explanation form is used to document information relevant to the conduct of the study that is not captured on other study forms (eg, explanation for missing variables or explanation for using a different metric than the one specified in the study summary). This form also includes a column for sites to indicate which method they used to obtain the data (ie, hand extraction for viral load only vs downloaded).

### Data Transfer

Data transfer will occur annually as site data extracts are uploaded and processed. Dropbox Business software will be utilized to allow for the transfer of relevant data files from sites to the REC and from the REC to the CM protocol leads and analytic core (AC) that are encrypted and meet all standards for Health Insurance Portability and Accountability Act (HIPAA) compliance. The Dropbox Business program and its rigorous HIPAA-compliant system for managing the sharing of individual folders will allow for the development of permissions that restrict access to each file to site-specific personnel, protecting participant confidentiality. The system uses a combination of user authentication, file audit trails, device and user permissions, emergency access protocols, and login/logoff checks to manage the integrity of files. The Dropbox Business program encrypts each file uploaded onto the Dropbox servers and then, with the appropriate permissions via *keys*, allows users to download and unencrypt the file on their local machine. This means that all files stored on Dropbox are completely encrypted such that anyone without the appropriate *key* on their local machine will be unable to open the file after downloading it—this process can be done live through shared folders or through Web-based file delivery (similar to a file transfer protocol delivery). To upload, a user drags and drops files to the appropriate folder or selects them via the menu, just as they normally would. As Dropbox Business will be used for data storage and the software employs secure encryption upon upload and Secure Sockets Layer (SSL) for data transmission, device encryption is not required at the sites.

Regarding Dropbox Business and encryption, the key features of Dropbox Business (a HIPAA-compliant cloud encryption software) are as follows:

1. Dropbox employs file-level encryption on devices and the cloud with Advanced Encryption Standard 256-bit encryption and uses SSL for transmission.
2. Any file placed in Dropbox is automatically encrypted. As Dropbox synchronizes only encrypted versions of files, the data are consistently protected.

Data transfer will occur annually, as site data extracts are uploaded and processed.

### Data Storage

Dropbox Business will be the primary method of data storage, and data files will be uploaded and downloaded securely by the REC, CM protocol lead and colead, and AC. Data files will not be saved on local machines under any circumstances. Regarding analyses, data will be analyzed by the CM protocol team and the AC using secure computer systems.

### Data Management

The protocol lead and colead will provide data management support for the AC, working closely with the clinical sites and the management core. One of their roles is specifically management of EHRs to develop a common data model across all 10 clinical sites.

The leads will ensure that analysis-ready datasets are regularly produced for the AC through ongoing data management. They will consult with the scientific team and with the AC to create a data management protocol, which details how finalized data will be structured. The data management protocol will be used in conjunction with the variable codebook to guide the data management process. Data will be exported from relevant sources and then imported into Statistical Analysis System (SAS Institute) and IBM Statistical Package for the Social Sciences (SPSS Inc). Using both SAS and SPSS, the leads will develop automated syntax files that will be used to clean data files by calculating scale scores, recoding variables, addressing missing data, and merging datasets together. Final datasets will be delivered to the AC in a preferred file format (SPSS, SAS, or CSV). Protocol leads will also provide ongoing support for cleaned data and perform additional data cleaning tasks as requested by the AC.

### Data Analysis Plan

Upon initial receipt of the data and at regular intervals, exploratory data analysis will be conducted to understand the breadth and specificity of available data. Descriptive statistics, including measures of central tendency, will be generated to identify outliers and track data consistency for future downloads. For the ATN as a whole, and also for sites individually, the proportion of individuals in each key stage of the cascade (retained in care, prescribed ART, and virally suppressed) will be described at baseline and over time.

The AC will perform longitudinal data analyses with advanced analytic procedures such as mixed-effects regression models, generalized estimation equations with a Poisson distribution, and log link function to model care retention based on both patient-related and clinical characteristics to identify the relevant predictors associated with dropout at any stage of the cascade. Moreover, these models will be utilized to identify trends and patterns of change over the course of data collection. We will also conduct additional analyses to assess baseline differences among sites as a function of previous training or participation in ATN studies. On the basis of the findings, the AC will provide suggestions to the SIU leadership, the protocol leads, sites, and community partners to support ATN protocols and development going forward.

#### Cost-Effectiveness Analysis Plan

The AC will specify costs of implementation for budgeting further scale up of SIU interventions but will also identify the incremental benefit of SIU interventions on cascade outcomes over time. The cost-effectiveness analysis for the study is designed to measure costs and consequences of changes in the implementation over the 48 months of study follow-up. Furthermore, the goal is to help inform the investigators of the economic consequences of the varying amount of resources used in the exploration, preparation, implementation, and sustainability components of the study [19]. The data collected through the CM study and the resulting EHR phenotypes for YLH will be used to construct episode costs from large billing databases that are relevant for modeling the cost-effectiveness of interventions aimed at YLH. The AC will use a previously developed cost utility model to estimate the cost per quality-adjusted life year over a 10-year time horizon expected from cascade outcomes of viral suppression and retention in care. As part of the identification of the larger cost-of-illness burden of cascade lapses for YLH, we will use the EHR phenotypes and archival data from Medicaid and/or privately insured populations to model the extent of cascade interruptions present in other practice settings. The data will be combined with the individual cost weights to estimate the variations in the economic burden that cascade disruptions for YLH place on US communities.

## Results

The first year of data extraction for the 2016 data was launched on December 4, 2017, in 10 nationwide clinic sites. The REC sent launch emails to each site with instructions for data formatting and transfer, as well as created individual Dropbox Business accounts for each site to ensure secure transfer and storage of data. Sites varied in the length of time to obtain and download data (from 2 months to 6 months post study launch). All sites successfully uploaded data by May of 2018; all sites were asked to download the following: age as of January 1, 2016, appointment dates, viral load, ICD-10 diagnoses codes, height, and weight. Additional variables were downloaded if available for electronic extraction, such as STI test results, cholesterol panel, race, and CD4 counts. In total, 70% of the sites provided data for all mandatory variables via electronic download. The remaining sites will provide data for all mandatory variables via electronic and hand extraction in the future. These variables may include various lab tests, gender, race/ethnicity, and weight. Therefore, we will have complete information for all mandatory variables from all sites for future data submissions. Although a few sites were not able to electronically download demographic information and specific lab results, the protocol team worked with each site to develop a plan for hand extraction of those variables.

The second year of data extraction for 2017 data was launched during October of 2018. The deadline for site data submission is scheduled for December of 2018. Subsequent data extractions will take place yearly in October through 2021. Data for the years of 2016 through 2021 will be available for data analysis as requested by specific SIU protocol team.

These uploads mark significant progress toward protocol goals to examine trends in the HIV treatment cascade. Through preliminary evaluation of uploaded data and correspondence with sites through the SIU Website’s Support Request Form system, the REC has learned valuable information that will facilitate the ATN’s extended knowledge on the pulse of the epidemic.

## Discussion

### Summary of Key Innovations

This study has several strengths. One is its sample size—by capturing data from 10 different clinic sites across the nation, it creates a robust dataset of YLH in the HIV treatment cascade. Moreover, the longitudinal design increases the amount of data available across the 10 sites and allows tracking youths’ progress over time, including an understanding of those who enter or exit the system between 2016 and 2021.

The longitudinal data also allow the SIU scientific team to track the impact of SIU clinic-based interventions, including ATN 146, Tailored Motivational Interviewing Implementation Intervention [20]. The EHR is beneficial as it reduces secondary data entry error when creating data files shared between sites and SIU personnel and does not rely on youth self-report of health. This is especially crucial for YLH, who may not yet be familiar with the details and nuances of their health information. With EHR data, we are able to obtain data that are specific and verified and gathered relatively quickly in large quantities. With the development of the phenotypes, we will be able to extend our study findings to identify lapses in the treatment cascade for YLH using large national databases. This process will permit the enumeration of this patient phenotype nationwide and the description of related annual economic costs of care. This will enable us to identify meaningful variations between communities that can then serve as the basis for targeting interventions.

Finally, the method through which we receive data is also secure and safe for participants. All datasets are deidentified at the clinic sites thereby minimizing risks to breach of confidentiality. Using Dropbox Business further secures patient data, as the service encrypts all data and is HIPAA compliant.

### Limitations and Conclusions

Despite significant benefits and innovation, study limitations exist. First, although there are 10 ATN clinic sites participating in this study, the majority of them are located in the Eastern part of the United States. There are only 2 sites west of Memphis and both (Los Angeles and San Diego) are in California. As a result, YLH who live in other parts of the United States, mainly the Midwest, Southwest, and West, are not included in this study. In addition, all clinics are situated in major cities. YLH living in smaller cities or in rural areas are also excluded. This potentially biases the sample, as YLH in nonurban areas experience different barriers to accessing and staying in care. For instance, those in urban areas may have greater access to public transportation and do not have to rely on cars or parking to visit a clinic. In addition, those in nonurban areas may have fewer clinics nearby, experience greater stigma from their community, or receive less structural support (in the form of policy or provider availability) for HIV-related care. Finally, the ATN clinics themselves are located within major academic research institutions. This suggests that the providers have more funding and support, as well as greater ability to adopt new evidence-based practices, than those without university support. As a result, the outcomes from this study will likely represent the best-case scenario for YLH in treatment. However, this limitation will be modified somewhat because of our plan to use the CM-developed EHR phenotypes to document cascade variations among communities represented by large archival databases, such as data from Medicaid and private insurers. Although such assessments will have errors and omissions at the level of individual patients, these analyses will provide important data at the population level. Even with the extended modeling, our findings will still be limited by the fact that the YLH in our CM study and by extension in the large national databases already know their status and have entered the HIV treatment cascade. There are many YLH who are still unaware of their sero-status or may not have the means or ability to seek care. This particular study is only able to investigate YLH who have already engaged in care in some way.

Despite these limitations, this study has the capacity to provide a wealth of crucial data for improving our understanding of YLH and their engagement in the HIV treatment cascade. Specifically, data from this study will indicate patterns of youths’ engagement in care across 10 different clinic sites and the manner in which this population changes over the course of 6 years. This research has the ability to provide high-quality data on trends in the HIV treatment cascade; specifically, how many of those retained in care and receiving ART adhere to their treatment plan and achieve viral suppression. Likewise, it will be possible to determine who falls out of treatment, providing clinics and policymakers critical information for how to better support these communities. These data will also be crucial for measuring the effect MI has on YLH outcomes over time.

## Abbreviations

AC: analytic core
ART: antiretroviral therapy
CM: cascade monitoring
CPT: Current Procedural Terminology
EHR: electronic health record
HIPAA: Health Insurance Portability and Accountability Act
ICD-10: International Statistical Classification of Diseases and Related Health Problems-10th revision
MI: motivational interviewing
MSM: men who have sex with men
PrEP: pre-exposure prophylaxis
REC: Recruitment and Enrollment Center
SIU: Scale It Up
SRV: subject recruitment venue
SSL: Secure Sockets Layer
STI: sexually transmitted infection
YLH: youth living with HIV
